# Exploring the mechanisms of neurotoxicity caused by fuzi using network pharmacology and molecular docking

**DOI:** 10.3389/fphar.2022.961012

**Published:** 2022-08-30

**Authors:** Junsha An, Huali Fan, Mingyu Han, Cheng Peng, Jie Xie, Fu Peng

**Affiliations:** ^1^ Key Laboratory of Drug-Targeting and Drug Delivery System of the Education Ministry and Sichuan Province, Sichuan Engineering Laboratory for Plant-Sourced Drug and Sichuan Research Center for Drug Precision Industrial Technology, West China School of Pharmacy, Sichuan University, Chengdu, China; ^2^ State Key Laboratory of Southwestern Chinese Medicine Resources, Chengdu University of Traditional Chinese Medicine, Chengdu, China; ^3^ College of Life Science, Sichuan Normal University, Chengdu, China

**Keywords:** fuzi, neurotoxicity, network pharmacology, molecular docking, aconitine

## Abstract

Safety has always been an important issue affecting the development of traditional Chinese medicine industry, especially for toxic medicinal materials, the establishment of risk prevention and control measures for toxic herbs is of great significance to improving the use of traditional Chinese medicine in clinical. Fuzi is a kind of traditional Chinese medicine and its toxicity has become the most important obstacle of limit in clinical using. In this paper, network pharmacology and molecular docking technology were used to analyze the main toxic components of Fuzi, the key targets and the mechanism of neurotoxicity. We carried out CCK-8 and WB assays, and detected LDH release and SDH activity. It was verified that aconitine caused neurotoxicity through a variety of pathways, including MAPK signaling pathway, pathways related to Akt protein, destruction of cell membrane integrity, damage of mitochondrial function affecting energy metabolism and apoptosis. What’s more, this study confirmed that aconitine could produce neurotoxicity by promoting apoptosis of hippocampus neuron and decreasing its quantity through Nissl Staining and TUNEL assay. This paper found and confirmed multiple targets and various pathways causing neurotoxicity of Fuzi, in order to provide reference for clinical application and related research.

## Introduction

Aconiti Lateralis Radix Praeparata (Fuzi in Chinese), known as Chinese aconite, Chinese wolfsbane and monkshood, is the lateral root of *Aconitum Carmichaelii* Debeaux (Zhao et al., 2020). Fuzi is a traditional Chinese medicine in China, which was recorded in the *Shennong herbal Scripture*. In the history of clinical use for thousands of yeas, numerous classical prescriptions such as four inverse soup, zhenwu decoction, and mahuang fuzi xixin decoction have emerged. As for today, modern research have shown that Fuzi has a wide range of pharmacological effects and is extensively used in treatment of cardiovascular diseases, rheumatism arthritis, neuropathic pain and bronchitis (Yang et al., 2018). Although Fuzi has promising therapeutic effects, its toxicities are frequently observed, like cardiac toxicity, neurotoxicity, hepatotoxicity, nephrotoxicity and so on (Chan, 2009). The current research about for cardiac toxicity of Fuzi is relatively wide, but the mechanism of its neurotoxicity still needs further research.

In 2007, the concept of “network pharmacology” was first proposed (Hopkins, 2007), in the same year, Chinese scholars used the biological network to study the traditional Chinese medicine prescriptions. In recent years, the research filed of systems biology has greatly advanced, as a result, the application of network pharmacology in traditional Chinese medicine has been developed and has a series of achievements (Li and Zhang, 2013; Zhang et al., 2019).

**FIGURE 1:**
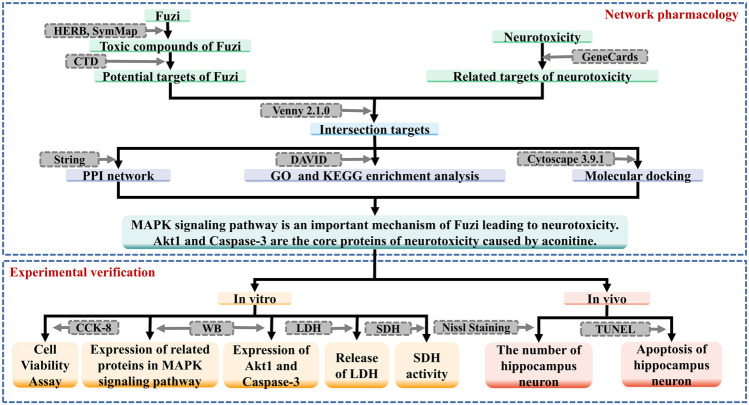
The workflow of this study.

In this study, on the basis of related literature reports, we aim to research the mechanism of neurotoxicity of Fuzi, using network pharmacology and molecular docking method, in order to provide reference for clinical application and related research. The workflow of this study is shown in Figure 1.

## Materials and methods

### Network pharmacology research

#### Screening the toxic compounds of fuzi and their targets

HERB database (http://herb.ac.cn/) and SymMap database (http://www.symmap.org/) were used to get all active compounds of Fuzi. Then, we retrieved the targets associated with active compounds from CTD database (http://ctdbase.org/), and our filter was set to “Interaction Count >1”. We collected all the targets, deleted duplicates and got candidate toxic compounds of Fuzi and potential target genes.

#### Predicting the targets of neurotoxicity

We searched potential targets of neurotoxicity by using “toxic encephalopathy” and “nerve toxicity” as the key words in GeneCards database (https://www.genecards.org/) and set “Relevance score ≥10” as a filter. Then the results of the two were combined and duplicated targets were removed in order to get potential targets of neurotoxicity.

#### Prediction of candidate targets and construction of PPI network

We used Venny 2.1.0 database (https://bioinfogp.cnb.csic.es/tools/venny/) to match the candidate targets of toxic compounds and the potential targets of neurotoxicity, and intersection target genes were the potential targets in the toxic effect of Fuzi on nerve.

All the targets were uploaded to String database (https://cn.string-db.org/) to build the PPI network interaction. Cytoscape 3.9.1 was used to construct and visualize the PPI network. CytoHubba, a network topology analysis plug-in in Cytoscape, was used for analyzing topology parameters of each target. The value of “Degree” was used as a reference for the importance of the core targets.

After that, we put the candidate toxic compounds of Fuzi and the corresponding 10 core targets into Cytoscape 3.9.1 to construct the relationship network between candidate toxic compounds of Fuzi and the corresponding 10 core targets.

#### GO biological process and KEGG pathway enrichment analysis

Intersected target genes were uploaded to DAVID database (https://david.ncifcrf.gov/) for performing GO biological process and KEGG pathway enrichment analysis (*p <* 0.05). GO enrichment analysis includes Biological Process (BP), Molecular Function (MF), and Cellular Component (CC) analysis. KEGG is a bioinformatics resource for mining significantly altered metabolic pathways enriched in the gene list. GO and KEGG pathway analysis were visualized using the R programming language.

#### Molecular docking

PubChem database (https://pubchem.ncbi.nlm.nih.gov/) was used to download the SDF format of key toxic compounds, and OpenBabel software was used to transfer into MOL2 format. The 3D structure of the protein was downloaded in PDB database (http://www.rcsb.org/).

Then, we imported the search results into AutoDock Tool software. The target protein was used as a receptor with the water molecule removed and nonpolar hydrogen added. The key toxic compound was used as a ligand, and the Grid box coordinated and size were set according to the target protein. At last, we selected the binding conformation with the lowest free binding energy by using AutoDock Vina and imported it into PyMol software for visualization.

### Experimental validation

#### Reagents

**FIGURE 2:**
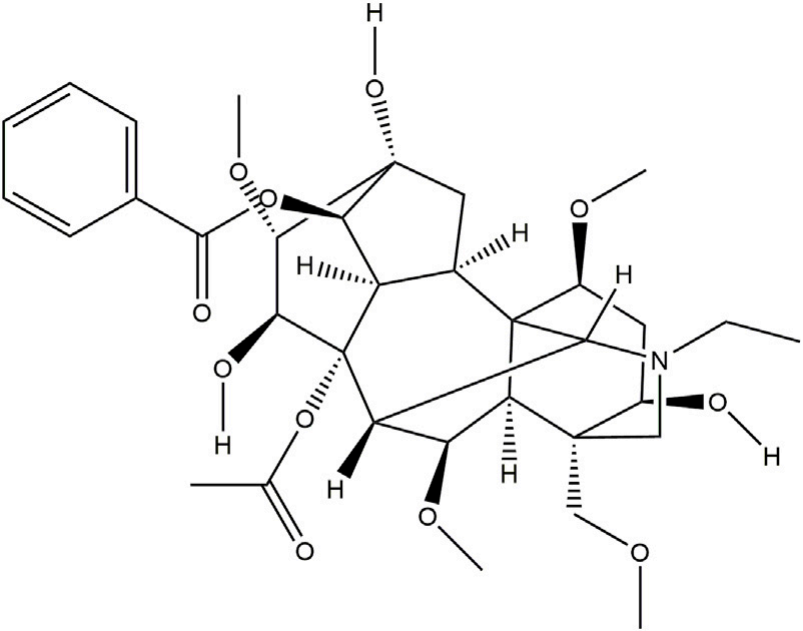
Chemical structure of aconitine.

Aconitine (purity ≥98%), the chemical structure is shown in Figure 2, was obtained from Chengdu Must Bio-Technology Co., Ltd. (Chengdu, China).

Dulbecco’s Modified Eagle’s Medium (DMEM) was purchased from Hylone (Logan, Utah, United States). 10% Fetal Bovine Serum (FBS) was obtained from GIBCO (NY, United States). The Cell Counting Kit-8 (CCK-8) was bought from Sigma-Aldrich (Nevada, United States). Penicillin-Streptomycin Solution was obtained from Allcare Biomedical Development (Qingdao, China). The primary antibodies against p-MAPK, MAPK1, p53, p-Akt, Akt, Caspase-3 and GAPDH were purchased from Cell Signaling Tech. (MA, United States). Trypsin, Tris-base, glutathione (GSH), acrylamide, SDS, AP, THMED and other reagents were obtained from Amresco (United States). Nissl Staining Solution and LDH Assay Kit were bought from Beyotime (Shanghai, China). SDH Assay Kit was obtained from Nanjing Jiancheng Bioengineering Institute (Nanjing, China). The *In Situ* Cell Death Detection Kit was purchased from Roche (Basel, Switzerland). Alcohol and xylene were obtained from SINOPHARM (Beijing, China). And paraformaldehyde was purchased from Sangon Biotech (Shanghai, China).

#### Cell culture

SH-SY5Y cells were obtained from cell bank in Shanghai Institutes for Biological Sciences (SIBS, CAS). They were cultured in DMEM medium supplemented with 10% (v/v) FBS and 1% (v/v) penicillin-streptomycin solution, and maintained at 37°C in a humidified chamber with 5% CO_2_.

#### Cell viability assay

Cell viability was measured using CCK-8 assay. SH-SY5Y cells were seeded into 96-well plates in the concentration of 5×10^3^ cells per well. After incubating in a cell incubator overnight, SH-SY5Y cells were exposed to aconitine in concentrations of 0, 25, 50, 100, 200, 300, 400 μM for 24 and 48 h. The wells added amount of medium is the blank group. Then, 10 μL of CCK-8 solution was added to each well of the plate. After 1 h of incubating, we measured the optical density (OD) at 450 nm wavelength with a microplate reader. Each experiment was repeated three times. (Cell viability = [(OD_Experiment group_ - OD_Blank_)/(OD_Control group_ - OD_Blank_)]×100%)

#### Western blot assay

SH-SY5Y cells (5×10^5^ cells/well) were seeded into 6-well plates and were exposed to aconitine in concentrations of 0, 100, 200, 400 μM for 24 h. Cell proteins were extracted after treatment with RIPA lysis buffer. The BCA method was used to calculate the content of protein. According to the relative molecular mass of target protein, we prepared 10% sodium dodecyl sulfate polyacrylamide gel electrophoresis (SDS-PAGE). An equal amount of the protein (20 μg per sample) was loaded onto SDS-PAGE for separation and transferred onto polyvinylidene difluoride (PVDF) membranes. Then we used 5% BSA for 1 h to block the membranes and used the primary antibodies including anti-p-MAPK, anti-MAPK1, anti-p53, anti-p-Akt, anti-Akt, anti-Caspase-3 and anti-GAPDH overnight at 4°C. After washed with TBST three times and further incubated with HRP-conjugated secondary antibodies for 1 h at room temperature, the protein bands were detected by the CheniDoc MP Imaging System.

#### Detection of lactate dehydrogenase release

SH-SY5Y cells were incubated in 96-well plates and exposed to aconitine in concentrations of 0, 25, 50, 100, 200, 300, 400 μM for 24 h. Then, 10 μL of Lysis solution was added to each well of the plate to assess maximum LDH release. We collected supernatants after centrifugation at 400 *g* for 5 min and transferred 120 μL of the supernatant from each well to a new assay plate. Finally, we added 60 μL of the detection solution to each well and recorded absorbance at 490 nm after 30min of incubating. Experiments were repeated three times.

#### Detection of succinate dehydrogenase activity

SH-SY5Y cells were incubated in 6-well plates and exposed to aconitine in concentrations of 0, 100, 200, 400 μM for 24 h. Then we collected supernatants after centrifugation. And the SDH activity was measured using SDH Assay Kit according to the manufacturer’s protocol. Experiments were repeated three times.

#### Animals and treatment

Twenty rats of SPF grade were used in this study. All rats were about six weeks old and divided into two equal groups for different experimental analysis. Each rat in group I (experimental) was given intraperitoneal injection (*i.p.*) of aconitine (1 mg/kg), while each rat in group II, which served as control, received an equal volume of normal saline.

The treatment was last for one week. Then, the rats were sacrificed. We quickly removed the brain tissue. Tissues for examination were fixed and preserved in paraformaldehyde, processed and trimmed, embedded in paraffin, and sectioned to a thickness of 5 μm.

These studies were conducted in compliance with the “Guide for the Care and Use of Laboratory Animals” (National Research Council (US), 2011).

#### Nissl staining experiment

The brain sections were dewaxed, rehydrated and washed with distilled water three times. Then we used Nissl Staining solution to stain the sections for 5 min and used distilled water to wash them twice. After being stained, the sections were dehydrated with 95% ethanol for 2 min (twice) and xylene for 5 min (twice), and then fixed using neutral gum. The number of hippocampus neuron was visualized with an optical microscope at 40 × and 100 ×  magnification. Then we used ImageJ software to count normal nerve cells.

#### TUNEL assay

Apoptosis cells were measured by using *In Situ* Cell Death Detection Kit. After being dewaxed, rehydrated and washed with distilled water three times, the sections were stained according to the manufacturer’s instructions and apoptotic cells were stained brown due to the binding of dUTP enzyme to their fragmented DNA. We used an optical microscope at 40× and 100 × magnification to observe apoptosis of hippocampus neuron. Then, we used ImageJ software to calculate the number of apoptotic cells.

#### Statistical analysis

All statistical analyses were preformed using GraphPad prism 8.0. Differences in multiple groups were analyzed by ANOVA and *p* < 0.05 was considered statistically significant.

## Results

### Toxic compounds of fuzi and their targets

We used HERB and SymMap database to predict the toxic compounds of Fuzi, and a total of 177 active compounds were retrieved. All the active compounds were input into CTD database for compound retrieval, and 22 candidate toxic compounds of Fuzi were obtained. After we combined the targets of all toxic compounds and deleted the duplicates, a total of 1810 corresponding targets were obtained. Then toxic compounds with corresponding targets greater than 10 were selected as candidate core toxic compounds (Table 1).

**TABLE 1:** Basic information of the core toxic components of Fuzi.

Ingredient name	CAS id	Molecule weight	OB score	Targets
Uracil	302-27-2	112.09	42.5256	1602
Palmitic Acid	67701-02-4	256.42	19.2966	216
Aconitine	302-27-2	645.74	7.9514	57
Linoleic Acid	60-33-3	279.40	-	39
Honokiol	35354-74-6	266.33	60.6694	34
14-Deoxy-11,12-Didehydroandrographolide	42895-58-9	332.43	13.6003	16
Juglone	481-39-0	174.15	25.7430	15
Magnolol	528-43-8	266.30	69.1927	11

### Targets of neurotoxicity

In the GeneCards database, 252 potential targets were searched by using “toxic encephalopathy” as the key word, and 290 potential targets were searched by using “nerve toxicity” as the key word. All the collected target genes were merged and duplicated. Then we obtained 474 potential targets of neurotoxicity.

### Prediction of candidate targets and construction of PPI network

**FIGURE 3:**
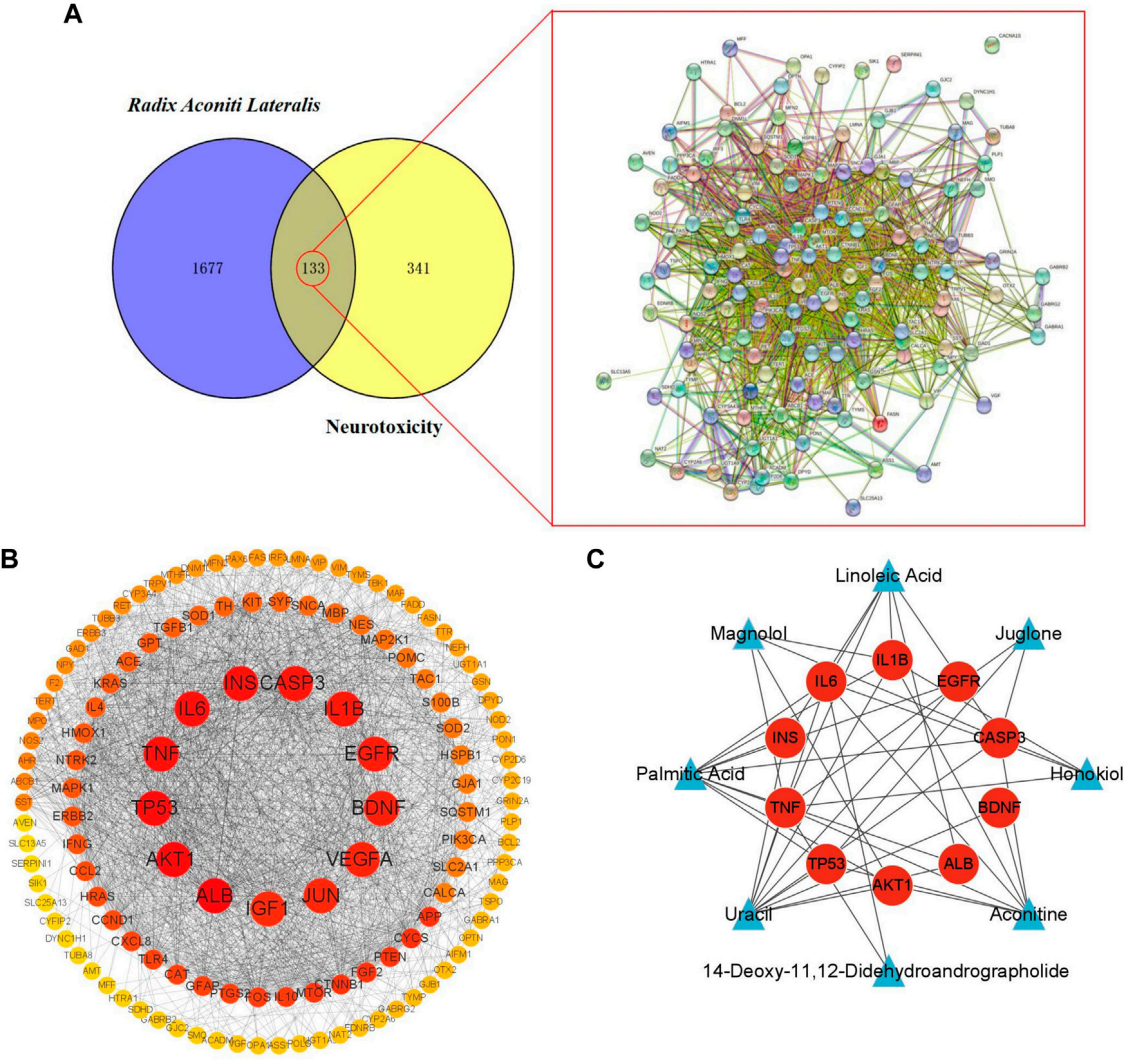
Target screening and establishment of PPI network. **(A)** Potential targets of neurotoxicity caused by Fuzi and PPI network. **(B)** PPI network of neurotoxicity targets caused by Fuzi. (The color of the node is marked from red to yellow according to the Degree value in descending order.) **(C)** Relationship network between candidate toxic compounds and core targets of Fuzi. (Blue triangles represent candidate toxic compounds and red circles represent core targets).

Matching the candidate targets of toxic compounds and the potential targets of neurotoxicity, 133 genes were selected as potential targets in the toxic effect of Fuzi on nerve. Then, String database was used to identify the PPI network of the 133 intersection target genes, as shown in Figure 3A.

We uploaded the PPI network in the figure above to Cytoscape 3.9.1 and used the Network Analyzer function in the software to analyze the topology parameters of each target (Table 2).

**TABLE 2:** Topological parameters of candidate targets.

Gene	Degree	Gene	Degree	Gene	Degree	Gene	Degree	Gene	Degree
ALB	174	HRAS	102	PIK3CA	62	MAF	36	EDNRB	18
AKT1	168	CCL2	100	SQSTM1	62	TBK1	36	UGT1A9	16
TP53	160	IFNG	98	CALCA	60	UGT1A1	34	NAT2	16
IL6	156	ERBB2	94	ABCB1	58	FASN	34	VGF	14
TNF	156	MAPK1	94	SST	58	TTR	34	OPA1	14
INS	156	NTRK2	94	AHR	58	FADD	34	ASS1	14
CASP3	146	KRAS	90	MPO	56	NEFH	34	POLG	14
IL1B	144	HMOX1	90	NOS2	56	DPYD	32	ACADM	12
EGFR	136	IL4	90	F2	54	GSN	32	SDHD	10
BDNF	134	GPT	86	TERT	54	PON1	30	SMO	10
VEGFA	130	ACE	86	NPY	52	NOD2	30	GABRB2	10
JUN	126	TGFB1	82	ERBB3	50	CYP2D6	28	GJC2	10
IGF1	124	KIT	80	RET	50	CYP2C19	28	HTRA1	8
CYCS	120	SYP	80	GAD1	50	GRIN2A	26	MFF	8
APP	120	SOD1	80	TUBB3	50	BCL2	24	CYFIP2	6
CTNNB1	118	TH	80	CYP3A4	48	PLP1	24	AMT	6
PTEN	118	SNCA	78	TRPV1	48	TSPO	22	DYNC1H1	6
FGF2	118	MBP	74	MTHFR	46	PPP3CA	22	TUBA8	6
MTOR	116	NES	72	MFN2	42	MAG	22	SLC25A13	4
IL10	110	MAP2K1	68	DNM1L	42	AIFM1	20	SIK1	4
PTGS2	108	POMC	66	FAS	42	GJB1	20	SLC13A5	2
FOS	108	TAC1	64	PAX6	42	OTX2	20	AVEN	2
CAT	106	SOD2	64	LMNA	40	OPTN	20	SERPINI1	2
TLR4	106	S100B	64	VIM	40	GABRA1	20	EDNRB	18
GFAP	106	SLC2A1	62	IRF3	40	GABRG2	20	UGT1A9	16
CXCL8	104	GJA1	62	VIP	40	TYMP	20	NAT2	16
CCND1	102	HSPB1	62	TYMS	36	CYP2A6	18	VGF	14

The top 10 are selected as the core targets according to the ranking of Degree value, followed by ALB, AKT1, TP53, IL6, TNF, INS, CASP3, IL1B, EGFR and BDNF. The visualization results are shown in Figure 3B.

Subsequently, we used Cytoscape3.9.1 to construct the relationship network between candidate toxic compounds of Fuzi and the corresponding 10 core targets in Figure 3C, and selected uracil, palmitic acid and aconitine as the key toxic compounds of Fuzi for subsequent analysis according to Degree value.

### GO biological process and KEGG pathway enrichment analysis

**FIGURE 4:**
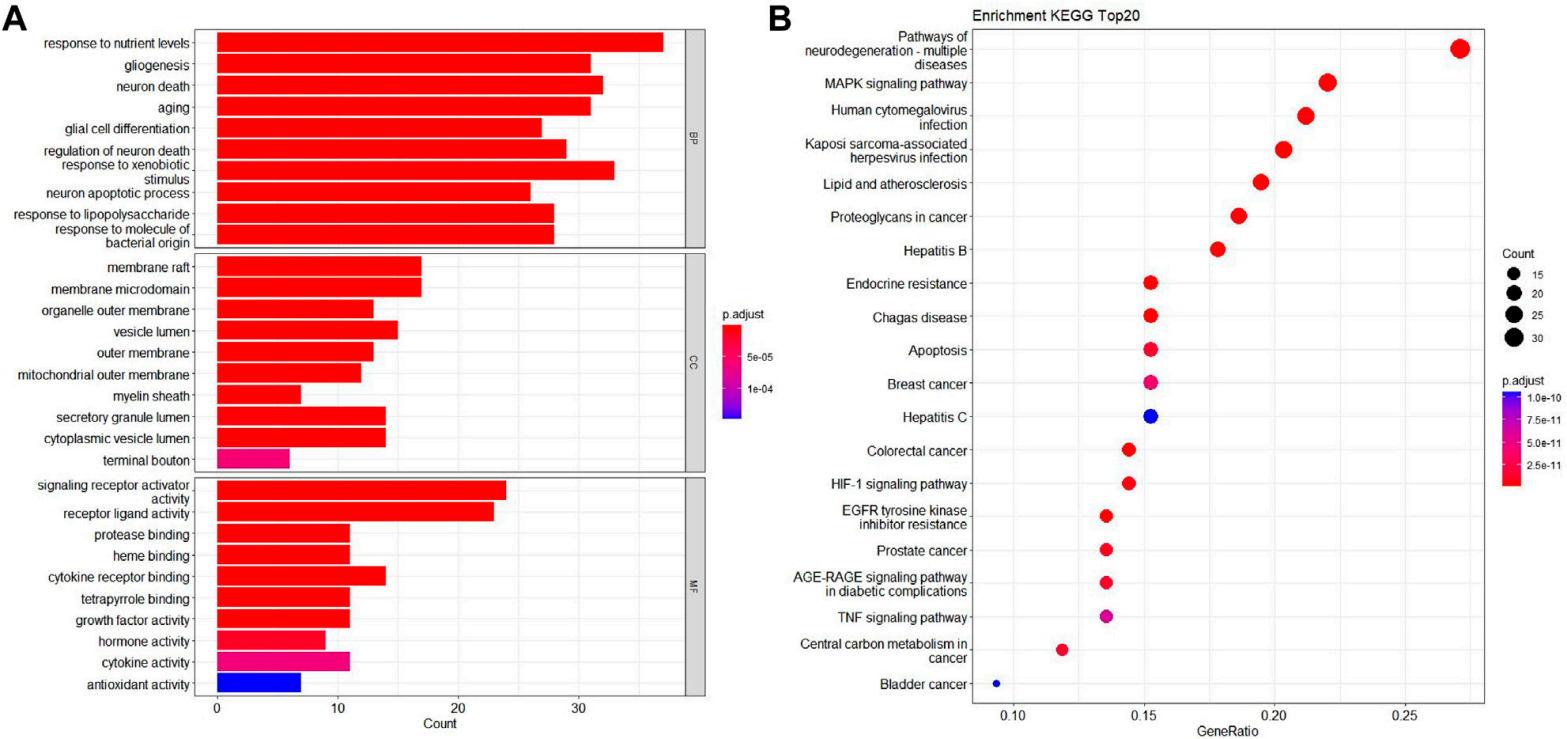
GO biological process and KEGG pathway enrichment analysis. **(A)** The bubble diagram of GO enrichment analysis of 133 intersection targets, including the top 10 significant enrichment terms of BP, CC and MF. **(B)** The bar plot diagram of KEGG pathway enrichment analysis of 133 intersection targets (top 20).

To explore the toxic mechanisms, we imported 133 intersection targets into DAVID database for GO and KEGG pathway enrichment analyses. A total of 184 GO functional items were obtained, including 124 biological processes (BP), which mainly involved response to nutrient levels, gliogenesis, neuron death and aging, etc. There were 18 cellular components (CC), mainly including membrane raft, membrane microdomain, organelle outer membrane and vesicle lumen, etc. It also included 42 molecular function (MF), focusing on signaling receptor activator activity and receptor ligand activity. The first 10 items of enrichment results were visualized according to *p*-value, as shown in Figure 4A.

We screened 170 major signaling pathways in KEGG pathway enrichment results. The high ranking enriched pathways involved pathways of neurodegenerative multiple disease, MAPK signaling pathway, HIF-1 signaling pathway, TNF signaling pathway, and various infection and tumor-specific pathways. Other pathways included lipid and atherosclerosis, Hepatitis B and Hepatitis C, endocrine resistance, apoptosis and so on. The most significant enriched 20 pathways in KEGG analysis were shown in Figure 4B.

Among them, MAPK1, TP53, TNF, EGFR, INS, CASP3, IL1B and BDNF in the core targets were all related to MAPK signaling pathway, which is an important mechanism of Fuzi leading to neurotoxicity.

### Molecular docking

In order to further verify the molecular mechanism of neurotoxicity of Fuzi, we chose uracil, palmitic acid and aconitine, the key toxic substances of Fuzi, to preliminarily simulate their binding to the core targets (Table 3).

**TABLE 3:** The binding energy between key toxic substances and core targets of Fuzi.

	ALB	AKT1	TP53	IL6	TNF	INS	CASP3	IL1B	EGFR	BDNF
Uracil	−4.6	−5.1	−4.4	−3.7	−3.1	−4.0	−3.8	−2.8	−3.6	−3.9
Palmitic Acid	−5.7	−3.9	−3.8	−3.9	−4.2	−3.6	−4.8	−4.1	−3.5	−3.9
Aconitine	−8.3	−7.4	−6.1	−5.7	−4.9	−4.1	−7.1	−6.3	−5.5	−6.7

It is generally believed that when the binding energy is less than −5.0 kJ mol^−1^, this compound has a good binding activity with the core target protein, while when the binding energy is less than −7.0 kJ mol^−1^, this compound has a strong binding activity with the core target protein (Hsin et al., 2013).

**FIGURE 5:**
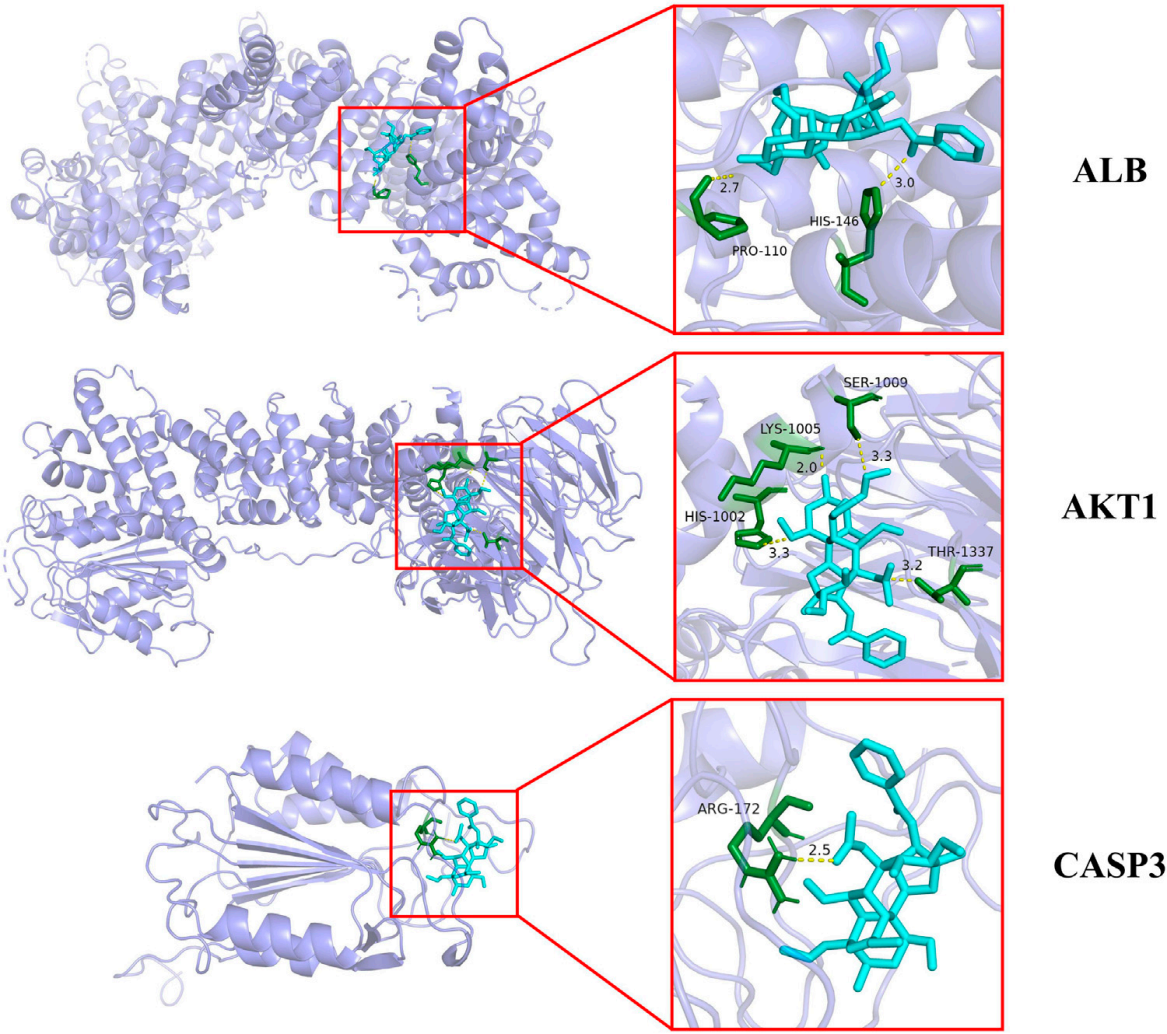
Molecular docking patterns of aconitine and core protein molecules. (The yellow lines represent the hydrogen bond interaction force, which is the main force promoting molecule binding with the active site.)

Among the key toxic substances of Fuzi, the binding energy of aconitine with ALB, AKT1 and CASP3 proteins were all less than −7.0 kJ·mol^−1^, indicating that aconitine was the most stable active ingredient. Figure 5 showed the docking diagram of aconitine with ALB, AKT1 and CASP3 protein molecules.

### Aconitine inhibits SH-SY5Y cell viability

**FIGURE 6:**
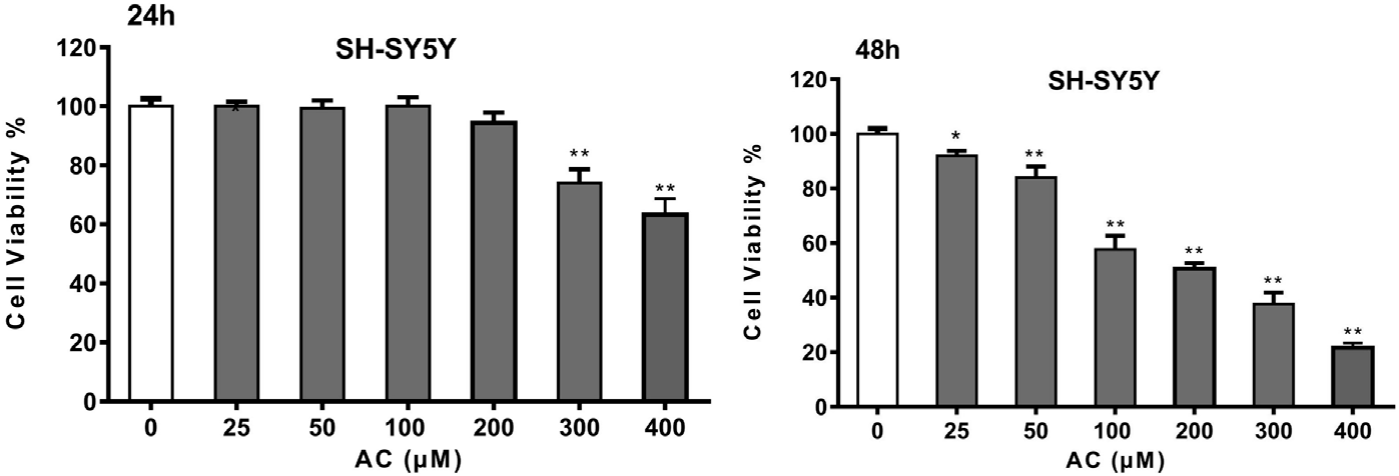
Cell Viability of SH-SY5Y cells after 24 and 48 h aconitine treatment. (*p < 0.05, **p < 0.01 versus control group.)

Network pharmacology analysis showed that aconitine was the main toxic substance causing neurotoxicity of Fuzi, so we further investigated the toxic effect of aconitine on SH-SY5Y cells. The results of CCK-8 assay showed that aconitine at 300 and 400 μM concentrations could decrease SH-SY5Y cell viability following 24 h treatment and aconitine at all the different concentrations (25, 50, 100, 200, 300 and 400 μM) significantly decreased SH-SY5Y cell viability after 48 h treatment, and the cell viability rate gradually decreased with the increase of concentration, indicating that aconitine had significant toxic effect on SH-SY5Y cells (Figure 6). Moreover, IC_50_ value of the CCK-8 is 271.4226 μM (48 h).

### Aconitine affects MAPK signaling pathway

**FIGURE 7:**
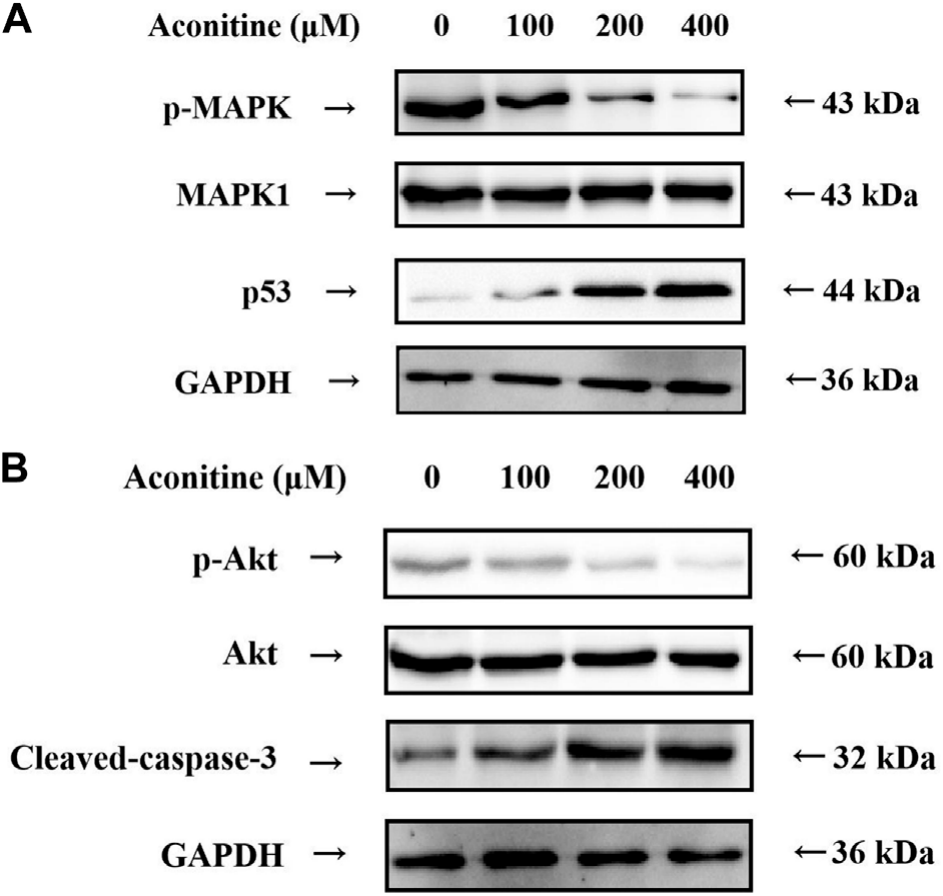
Western blot analysis after 48 h aconitine treatment. **(A)** Effects of aconitine on the protein secretion of MAPK pathway in SH-SY5Y cells. **(B)** Effects of aconitine on the secretory levels of p-Akt, Akt and Cleaved-caspase-3 in SH-SY5Y cells.

According to the results of the KEGG enrichment analysis, MAPK signaling pathway was an important mechanism of neurotoxicity caused by Fuzi. Therefore, we further evaluated the expression levels of p53, p-MAPK and MAPK1, which are the core target proteins in MAPK signaling pathway. As shown in Figure 7A, aconitine inhibited the expression of p-MAPK, while the expression of MAPK1 remained basically unchanged. Meanwhile, aconitine also promoted the expression of p53. These results suggested that aconitine can inhibit phosphorylation of MAPK and the toxic effect of aconitine on SH-SY5Y cells was related to MAPK signaling pathways.

### Aconitine affects the expression of the core proteins

Molecular docking results showed that Akt1 and Caspase-3 proteins were the core proteins of neurotoxicity caused by aconitine. Therefore, we assessed the expression level of p-Akt, Akt and Caspase-3 by western blot. As shown in Figure 7B, treatment of SH-SY5Y cells with aconitine (100, 200, 400 μM) led to apparent repression of phosphorylation level of Akt in a dose-dependent manner, with the expression of Akt remaining unchanged. What’s more, aconitine promoted the expression of Caspase-3. The results indicated that the mechanism of neurotoxicity induced by aconitine was associated with inhibition of Akt phosphorylation and promotion of apoptosis.

### Aconitine increases the release of LDH

**FIGURE 8:**
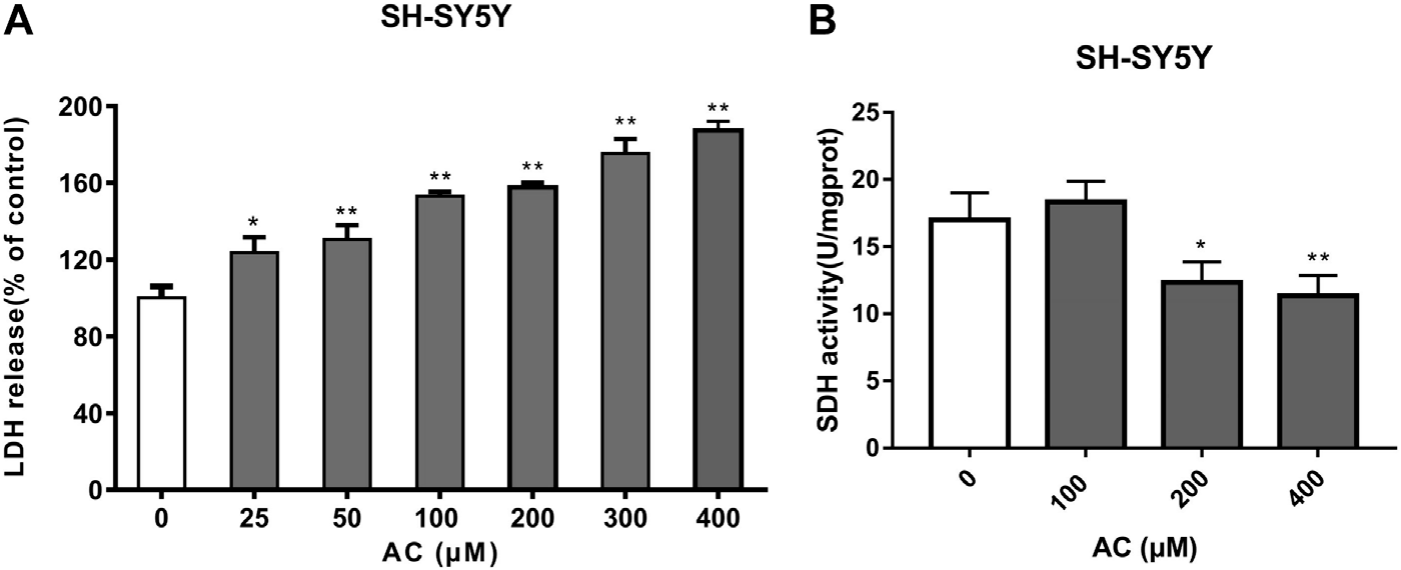
Effects of aconitine on mitochondrial function. **(A)** The release of LDH of SH-SY5Y cells after 24 h aconitine treatment. (*p < 0.05, **p < 0.01 versus control group.) **(B)** The SDH activity of SH-SY5Y cells after 24 h aconitine treatment. (*p < 0.05, **p < 0.01 versus control group).

LDH is a stable cytoplasmic enzyme and the release of LDH is a key feature of the plasma membrane damage. Compared with the control group, aconitine at different concentrations (25, 50, 100, 200, 300 and 400 μM) significantly increased the release of LDH in Figure 8A, which suggested that cell membrane damage was an important toxic effect of aconitine and might be a key pathway for other exogenous substances entering the cell.

### Aconitine reduces SDH activity

SDH, belonging to cytochrome oxidase, is the only multi-subunit enzyme integrated on the membrane in the TCA cycle. It can provide electrons for the respiratory chain of mitochondrial oxygen demand and productivity, and is a marker enzyme of mitochondrial. As shown in Figure 8B, aconitine at 200 and 400 μM for 24 h significantly decreased SDH activity. Under the action of aconitine, the activity of SDH in nerve cells decreased at high dose, and the dose-effect relationship was obvious.

### Aconitine reduces the number of normal hippocampal neurons

**FIGURE 9:**
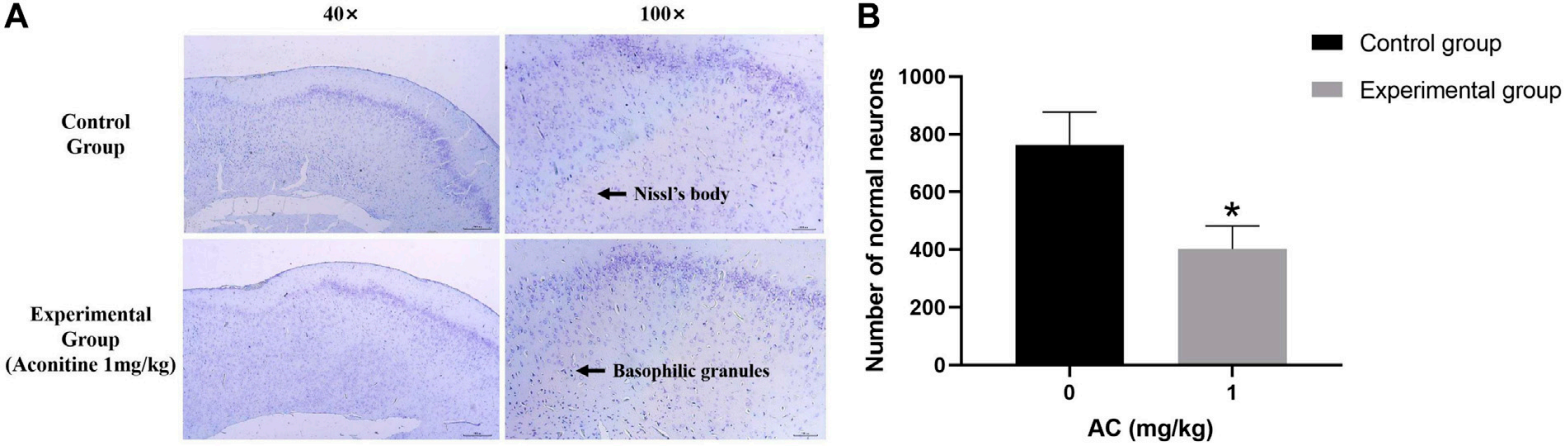
Treatment of aconitine for 7 days reduced the activity of hippocampus neurons. **(A)** Representative images magnified 40 and 100 times in hippocampus. **(B)** Quantitatively analyzed the number of Nissl’s bodies in control and experimental group (×100). (*p < 0.05 versus control group).

The Nissl Staining result is shown in Figure 9, we can observe that the structure of Nissl’s body in control group is clear, the basophilic granules distribute evenly and the number is more. However, the number of Nissl’s body in experimental group is less than the normal group, and there are a large number of basophilic granules gathering which is caused by numerous Nissl’s bodies dissolving.

### Aconitine induces apoptosis of hippocampus neurons

**FIGURE 10:**
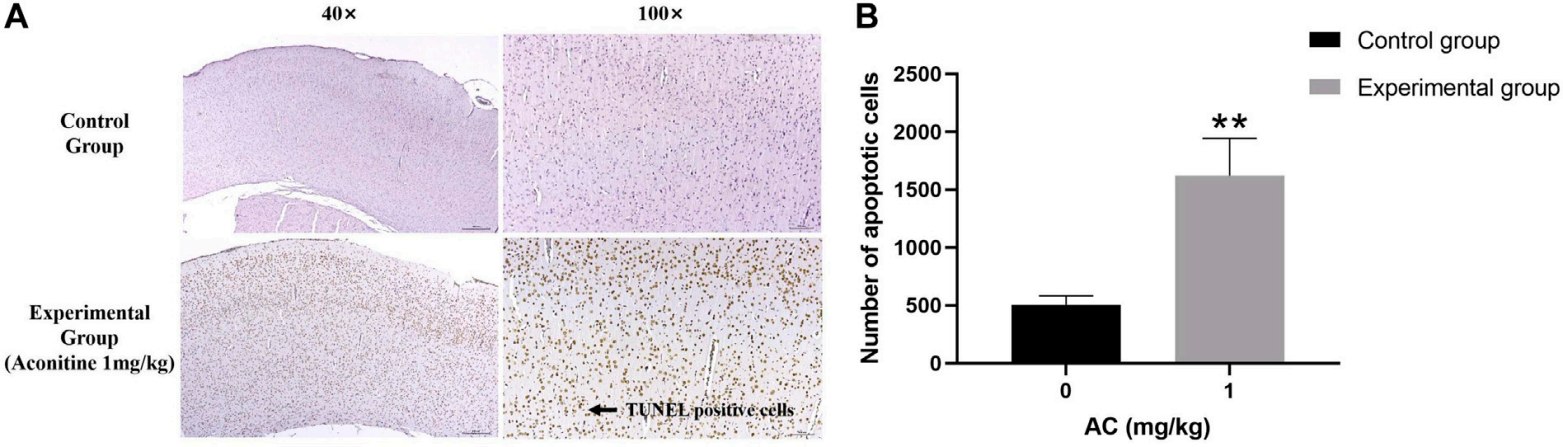
Treatment of aconitine for 7 days increased apoptosis of hippocampus neurons. **(A)** Representative images magnified 40 and 100 times in hippocampus. **(B)** Quantitatively analyzed the number of apoptotic cells in control and experimental group (×100). (**p < 0.01 versus control group.)

Compared with the control group, we can see a large number of TUNEL positive cells (brown) in the experimental group obviously. Its expression area is wider and its dyeing is heavier (Figure 10). The result illustrates that aconitine can promote apoptosis of hippocampus neurons.

## Discussion

Based on network pharmacology, molecular docking and experimental verification, this study systematically analyzed the potential mechanism of neurotoxicity caused by Fuzi, constructed the relationship network between candidate toxic compounds and neurotoxicity targets, and predicted the main toxic substances, potential targets and signaling pathways.

Network pharmacology analysis showed that uracil, palmitic acid and aconitine might play a crucial role in the neurotoxicity of Fuzi. Uracil is an important component in RNA, and fluorouracil, an antitumor drug with similar structure, has long been reported to have obvious neurotoxicity (Pirzada and AliDafer, 2000). Palmitic acid, as a long-chain saturated fatty acid, is an important component of blood lipids. Studies have shown that palmitic acid induces apoptosis by increasing oxidative stress in nerve cells, thereby producing neurotoxicity (Ng and Say, 2018). In addition, aconitine is considered to be the main toxic substance of Fuzi, Chuanwu and Caowu, and its toxic effects have been widely studied. The above existing research results are basically consistent with the prediction results, suggesting that the main toxic substances of Fuzi are closely related to its neurotoxicity.

The result of PPI protein interaction network analysis showed that the potential target genes for neurotoxicity of Aconite were ALB, AKT1, TP53, IL6, TNF, INS, CASP3, IL1B, EGFR and BDNF. AKT1 is the core target of PI3K/AKT signaling pathway. MAPK1, TP53, TNF, EGFR, INS, CASP3, IL1B and BDNF are related to MAPK signaling pathway. GO function and KEGG pathway enrichment analysis also confirmed that MAPK signaling pathway played an important role in the neurotoxicity induced by Fuzi.

Molecular docking results displayed that aconitine, the main toxic substance of Fuzi, exhibited good affinity with key targets ALB, AKT1 and CASP3, and the conformation of the binding site was stable. Based on the above results, the main toxic substance aconitine was selected for subsequent experimental verification, and its potential mechanism was discussed in depth.

The results of WB assay confirmed that aconitine affects the transmission of MAPK signaling pathway by inhibiting the MAPK protein phosphorylation and promoting the expression of p53. In KEGG PATHWAY database (https://www.genome.jp/kegg/pathway.html), we found that MAPK and p53 proteins are mainly involved in the classical MAPK pathway, JNK and p38 MAPK pathwayand p53 signaling pathway, and we suspected they are the main signaling pathways of neurotoxicity caused by Fuzi.

At the same time, the WB results showed that aconitine can inhibit the phosphorylation of the key protein Akt and promote the expression of Cleaved-caspase-3. Akt is the core protein of PI3K-Akt signaling pathway, and can control a variety of downstream signaling pathways, therefore, by adjusting the phosphorylation of Akt, Fuzi affects PI3K-Akt signaling pathway and controls various signaling pathways to cause neurotoxicity. And Cleaved-caspase-3, a kind of cysteine protease, is one of the key enzymes in the cell apoptosis pathways. Fuzi can increase its expression, and then promote the apoptosis of nerve cells in the resulting in neurotoxicity.

Aconitine can also increase LDH release and reduce SDH activity. The increase in LDH release suggests that aconitine can destroy the integrity of neuronal cell membrane, and the decrease in SDH activity suggests that aconitine leads to mitochondrial damage, while the damage of cell membrane and mitochondrial dysfunction are the characteristics of irreversible cell damage (Grimm, 2013). Aconitine may affect mitochondrial energy metabolism by inhibiting ATP production and aerobic respiratory function in nerve cells, and destroy the integrity of the cell membrane, leading to the entry of exogenous substances, thereby causing toxicity to nerve cells.

Mitochondrial dysfunction can lead to energy deficiency, which is an important mechanism for neurotoxicity (Liu et al., 2021). Early studies have found that mitochondrial dysfunction is associated with MAPK signaling pathway, leading to neuronal apoptosis and aggravating brain injury (Kleefstra et al., 2011; Gui et al., 2020; Manikanta et al., 2020). The relationship between mitochondrial function and MAPK signaling pathway needs further study. And appropriate targeted regulation of mitochondria may be a new direction to avoid neurotoxicity of Fuzi.

The hippocampus is the most vulnerable areas of the brain and plays an important role in learning function impairment, memory loss and cognitive dysfunction (Knierim, 2015). Apoptosis is the main cause of hippocampus damage. This research verifies that aconitine has toxicity to the nervous system. As shown by the results of Nissl Staining and TUNEL assay, we can find that aconitine produces neurotoxicity by promoting apoptosis of rat hippocampus neurons and reducing the number of neurons.

In fact, the mechanism of these pathways is not a single work, but the interaction influence causes neurotoxicity of Fuzi. However, these signaling pathways also play an important role in treatment in addition to inducing toxicity (Yang et al., 2020; Chen et al., 2022). We are unable to completely block the pathways to avoid the toxicity of Fuzi, and a more effective approach is to remove the toxic components by processing.

Earlier studies have demonstrated that the toxicity of Fuzi mainly derives from diester diterpenne alkaloids including aconitine (Singhuber et al., 2009), which has been confirmed by molecular docking in this article. In the clinic, we can boil Fuzi in water for a long period of time, transforming hypaconitine into monoester-diterpenoid alkaloids and finally into unesterified compounds, which has no toxicity and no influence on its pharmacological activities (Lu et al., 2010; Zhou et al., 2015).

In our study, some drawbacks should be noted. Our research only contains the toxic components that have been confirmed at present and whether there are other toxic compounds in Fuzi needs to be further studied. In addition, most of the Chinese herbal medicine plays a role in treatment by the form of oral, so its efficacy and toxicity are closely related to metabolites. Unfortunately, our research didn’t predict all the bioactive metabolites of Fuzi *in vivo* (Feng et al., 2021).

In recent years, gut microbiota has emerged as a new frontier to understand the development and progress of diseases, especially it can influence the development and diseases of the central nervous system along “microbiota-brain-gut axis” (Quigley, 2017; Strandwitz, 2018). Arachic acid, a composition of Fuzi, is reported to be able to modulate the composition of gut microbiota (Zhuang et al., 2017; Sun et al., 2021). And whether Fuzi can cause neurotoxicity by influencing the gut microbiota still needs to be further discussed.

## Conclusion

This research analyzed the potential mechanism of neurotoxicity caused by Fuzi through building the relationship network of “compounds - targets - neurotoxicity” using network pharmacology and molecular docking. The results showed that aconitine, the core toxic compound of Fuzi, caused neurotoxicity through multiple targets and multiple ways, including MAPK signaling pathway, pathways related to Akt protein, destroying cell membrane integrity, damaging mitochondrial function and affecting energy metabolism and cell apoptosis. At the same time, aconitine can promote apoptosis of hippocampus neuron and decrease its quantity, thus producing neurotoxicity. Our research provided reference for clinical application and related research.

## Data Availability

The datasets presented in this study can be found in online repositories. The names of the repository/repositories and accession number(s) can be found in the article/Supplementary material.
